# xIV-LDDMM Toolkit: A Suite of Image-Varifold Based Technologies for Representing and Mapping 3D Imaging and Spatial-omics Data Simultaneously Across Scales

**DOI:** 10.1101/2024.11.04.621983

**Published:** 2024-11-05

**Authors:** Kaitlin M. Stouffer, Xiaoyin Chen, Hongkui Zeng, Benjamin Charlier, Laurent Younes, Alain Trouvé, Michael I. Miller

**Affiliations:** 1 Center for Imaging Science, Johns Hopkins University, Baltimore,MD, USA.; 2 Department of Biomedical Engineering, Johns Hopkins University, Baltimore,MD, USA.; 3 Kavli Neuroscience Discovery Institute, Johns Hopkins University, Baltimore, MD, USA.; 4 Centre Borelli ENS Paris-Saclay, Gif-Sur-Yvette, France.; 5 Allen Institute for Brain Science, Seattle,WA, USA.; 6 IMAG, Université de Montpellier, CNRS, Montpellier, France.; 7 Department of Applied Mathematics and Statistics, Johns Hopkins University, Baltimore, MD, USA.

**Keywords:** image varifold, cross-modality mapping, multi-scale, omics data

## Abstract

Advancements in imaging and molecular techniques enable the collection of subcellular-scale data. Diversity in measured features, resolution, and physical scope of capture across technologies and experimental protocols pose numerous challenges to integrating data with reference coordinate systems and across scales. This resource paper describes a collection of technologies that we have developed for cross-modality 3D mapping for the alignment of transcriptomics at the micron scales of genes and cells to the anatomical tissue scales. Our collection of technologies include (i) an explicit censored data representation for the partial matching problem mapping whole brains to subsampled subvolumes, (ii) image-varifold measure norms for supporting nearly universal crossing of modality, (iii) a multi, scale-space optimization technology for generating resampling grids optimized to represent spatial geometry at fixed complexities, and (iv) mutual-information based functional feature selection. Collectively, these methods afford efficient representations of peta-scale imagery providing the algorithms for mapping from the nano to millimeter scales which we term cross-modality image-varifold LDDMM (xIV-LDDMM).

## 1 Introduction

Emerging technologies in spatial transcriptomics coupled with advancements in imaging over the last decade are continuing to burgeon the scale and scope of biological data that can be interrogated. The uniqueness in phenomenological angles each method offers coupled to choice of employment amidst different species, across different tissue windows, and under different experimental conditions afford incredible breadth of data concurrently with the much-needed depth and replicate numbers that are often limited in what remain resource-heavy endeavors. Nevertheless, both the sheer amount of data coupled with the diversity in measured features, resolution, and physical window of capture across technologies and experiments pose challenges to integrating data across them and with reference coordinate systems at coarser scales. This integration is necessary, however, for downstream analysis and fully gleaning what understanding these datasets in combination might elucidate.

With regard to magnitude, detections are occurring at increasingly high resolutions (e.g. individual mRNA at submicron scale) and covering measurements of increasingly large feature sets (e.g. 20,000 different genes in the full mouse genome) [1]. Technologies are typically categorized into those which are spot-based (e.g. SlideSeq, Visium) versus those which are image-based (e.g. MERFISH, BARseq). The former group prioritizes feature set size at the expense of achieving only near-single cell resolution while the latter prioritizes single and even subcellular resolution at the expense of reducing the measured feature set (e.g. < 1000 genes).

For instance, in a single mouse coronal section measuring 500 different genes with MERFISH technology [2, 3], typically on the order of 100 million mRNA transcripts might be detected. With sections taken at ever 100 micron and spanning about 65–75% of the mouse brain anterior to posterior, the total number of mRNA detections across the resulting 50 sections is on the order of 6 billion. Covering an area of about 1 mm^3^, representation of these 6 billion mRNA at even a 1*μ*m voxel grid resolution would thus require 300–400 billion voxels, without even affording complete differentiation of neighboring mRNA from one another.

With regard to diversity, functional measures vary with both scale and type, with subsets of possible features often selected per experiment given the relevance to the problem at hand. At the tissue scale, measures interrogate both structural properties of anatomy (e.g. MRI, CT) as well as function (e.g. fMRI) that have often been summarized in the form of regional atlases (e.g. Allen CCFv3 [4]). Spatial-omics technologies, for instance, operate across scales of molecules to cells, coupling measures from imaging, sequencing, and mass-spectrometry [5] to downstream segmentation and clustering processes [6] to generate either molecule- or cell-based datasets sporting molecule (e.g. gene, protein, metabolite) type or cell type. While many such technologies necessarily capture static assessments of organisms, technologies focused on epigenomics [7] or synapse activity [8] are often coupling such profiles over time to capture the dynamics of both interacting cells/molecules.

Molecular Computational Anatomy, born recently out of the theory developed for modeling anatomy at tissue scales, naturally accommodates the diversity in scales, scope, and features attributed to data captures across experiments and technologies. In the Molecular Computational Anatomy model, [9, 10] brain mapping follows the D’Arcy Thompson [11] program requiring a set of objects that we call varifold measures denoted here as μ∈ℳ, with a norm $\|\mu {\|}_{M}$ to measure closeness [9, 10]. Thompson’s brain mapping scheme defines transformations which we model here as diffeomorphisms, φ∈Diff, transforming one brain to the other.

Our molecular representations (varifold measures) at the micron scales are represented mathematically as a set of particles, each particle carrying a singular mass weight located in physical space and a conditional probability distribution over the feature space [12] of for instance, genes or cell types. The brain mapping problem estimating φ:μ↦φ⋅μ, aims to minimize the normed distance between brain objects $\mu ,\mu^{\prime }\in ℳ$, optimizing over diffeomorphic transformations between them:

(1) $$\underset{\varphi \in Diff}{inf}\;{\left\|\varphi \cdot \mu -\mu^{\prime }\right\|}_{M}^{2}\;.$$

We call this the image-varifold representation using the varifold norm [9, 10, 12] which generalizes to many types and diversity of features while efficiently representing the particular sampling schemes (e.g. regularized grids versus innate cell placement) of each technology. As measurements and atlasing occurs across many scales, the formalism naturally supports hierarchical representations from tissue to molecular scales [9, 10]. Our descriptions throughout carry appropriate spatial scales associated to the smoothing kernels which produce a sequence of successive approximations of greater and greater detail in the dimensions of space-scale and gene/cell feature denoted explicitly as $\mu_{\sigma_{k}}$ where k ranges over a collection of scales satisfying $\sigma_{1}>\sigma_{2}\ldots .$ We exploit this multi-scale representation demonstrating mappings between the sub-micron scales of the transcriptome to the millimeter scales of the CCFv3.

Furthermore, while most spatial transcriptomics technologies report measures in a 2D landscape, increasingly these planes are representative of single sections out of stacks of planes extracted within a 3D volume [2, 13] that need accordingly to be integrated with corresponding 3D volumetric images and atlases. We have previously described the framework of the image-varifold representation for particle representations of spatial-omic measurements [10, 12]. The focus of this resource paper is to solve the alignment problem at the complexity of fully 3D, billion transcript measurements [14]. Accordingly, new technologies are needed for accommodating variants in scope and region of tissue capture as well as the peta-scale complexity of the data when spanning 3 dimensions. We describe here the collection of technologies we have developed addressing these challenges with independent modules for censoring, scale-space optimized particle approximations, and optimized feature selection that can integrate with our cross-modality image-varifold based large deformation diffeomorphic metric mapping scheme (xIV-LDDMM, [12]) to achieve alignment across scales and modalities in 3D.

## 2 Results

### 2.1 Overview of Technologies

**Fig. 1:**
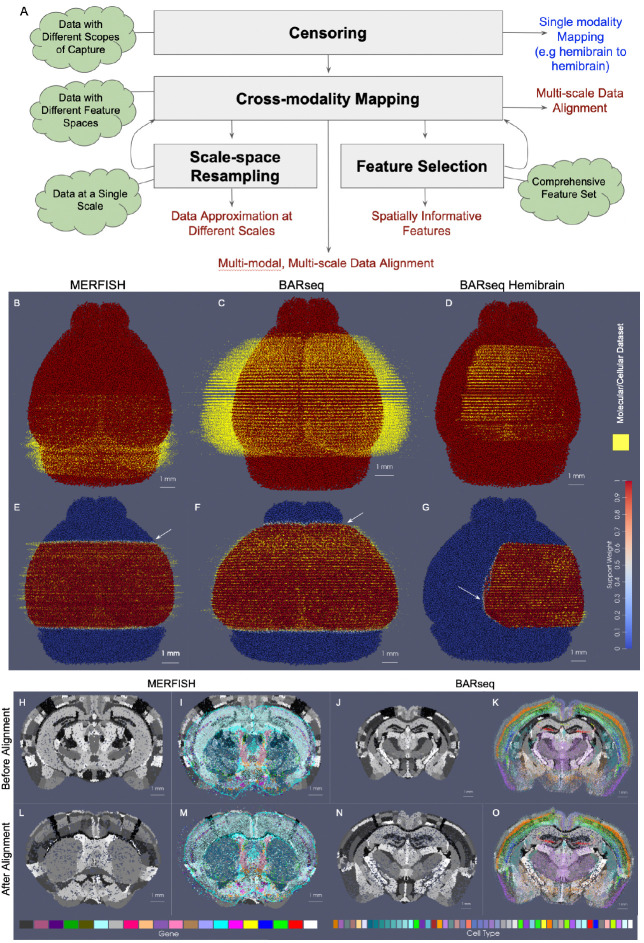
Summary of technologies and alignment of 3D atlas to molecular/cellular targets with automatic estimation of target tissue domain. A shows schematic of technologies (grey boxes) with specific input (green), output (red), and downstream uses (blue). B-G show estimated support weights in transforming whole brain CCFv3 to MERFISH and BARseq data. B-D show initial alignment of CCFv3 to parallel sections of MERFISH (B) or BARseq whole brain (C) or hemi-brain (D) data, with initial support weights in atlas set to 1 (red). E-G show transformed CCFv3 optimally aligned to molecular/cellular sections, with estimated support weights per particle. White arrows indicate smooth transition zone from within support (weight of 1) to outside support (weight of 0). Both CCFv3 and molecular/cellular targets depicted as 200 um approximations, with total particles numbering 68k (CCFv3), 95k (MERFISH), 163k (BARseq whole brain), 62k (BARseq hemi-brain). H-I and J-K show initial intersecting slice through CCFv3 aligning to single MERFISH (H,I) or BARseq (J,K) section of data. L-M and N-G show intersecting slice after alignment.

The specific modules described herein center on advancements including: (i) an explicit model for solving the partial matching problem mapping whole brain tissue scale atlases to partial volume censored targets, (ii) image-varifold norm for cross-modality mapping, (iii) scale-space approximation methods for optimized particle approximation of targets from molecular to tissue scales, (iv) mutual-information based feature selection technologies for optimized selections of genes/cell types for optimized sparse dimension reduction. As independent modules, we describe each in turn, but with an emphasis both on individual output and collective output as integration of these modules offers opportunities for refinement of alignment between modalities across scales (Figure 1A).

While current technologies afford 3D data collection as a series of 2D sectional data at high resolution, the scope and extent of tissue (3D volume) spanned by the series of sections delivered often varies with the experimental design. Additionally, spatial-omics techniques are still newly emerging and remain quite costly. Consequently, the vast majority of experimental designs often focus collection on brain subregions, delivering highly variable and partially sampled portions of target brains. This poses significant challenges to integration with tissue-scale atlases which cover the 3D volumes in their entirety, and is becoming only more prevalent as spatial-omics technologies are applied in settings of larger magnitude, such as primate brains, where measuring full or even hemi-coronal sections, is in itself, a challenge.

The partial subvolume censoring problem thus arises whenever aligning complete 3D tissue-scale atlases to partial volume captures, rendering it central to alignment problems for of spatial-omics. To solve this problem, we introduce in our first technology a spatial censoring function as part of the imaging model that punctures the Hilbert-space matching norm within the scope of the deforming atlas thereby optimizing alignment over the censored sample. We demonstrate the robustness of this technology for capturing rostral-caudal extent and medial-lateral extent of target data for sets of whole brain and half brain coronal sections of both MERFISH and BARseq data.

The core of our set of technologies is our second module comprised of a cross-modality mapping scheme that harnesses the image-varifold representation to model equivalently tissue-scale imagery and highly resolved molecular scale spatial-omics measurements. These representations are equipped with a norm that defines closeness with regard to feature and physical geometric similarity, and which we aim to minimize through estimation of both geometric (diffeomorphic) and feature-based associations that we term cross-modality image-varifold based large deformation diffeomorphic metric mapping (xIV-LDDMM).

Additionally, our mapping technologies, which are particle-based, include calculation of Hilbert space inner products which are quadratic in nature, placing us into a solution space that is staggeringly peta-scale in complexity. The magnitude of the sheer data that results from integration across sets of sections (e.g. 6 billion mRNA reads and order 1000 genes) present remarkable complexity challenges. Approximation and feature selection is not an afterthought but remains fundamental to the problem itself. Our third and fourth technologies address this complexity through reduction along both physical and feature axes, respectively. Specifically, our third technology generates a hierarchy of space-scale approximations, delivering the closest particle solution of a fixed complexity in the gene-cell type approximation metric to the target. Our fourth technology is an information-based technology for functional feature selection, where dimension reduction is achieved through selection of spatially varying genes/cell types that exhibit a high score based on mutual information.

We demonstrate each module of our set of technologies specifically in the setting of mapping the tissue-scale Allen CCFv3 atlas to cellular and molecular scale target serial sections generated through BARseq and MERFISH technologies, respectively.

### 2.2 Data sets: MERFISH and BARseq

The results described herein are from three different spatial transcriptomic datasets, including a published MERFISH dataset [2] with 56 coronal sections that cover most of the mouse brain except for the most rostral and caudal part, a newly collected BARseq dataset with 40 coronal sections that cover most of the mouse forebrain, and a set of 8 published BARseq datasets [15], each including 32 hemi-coronal sections that cover one hemisphere of the forebrain except the most rostral parts. The MERFISH dataset interrogated about 500 genes which were optimized for distinguishing all cell types across the whole brain, whereas all BARseq datasets interrogated 104 genes that were optimized for resolving cortical excitatory neuron types, with less emphasis for subcortical cell types. Hence, all three datasets are examples of censored subsets relative to the whole brain atlas.

Our results have focused on demonstrating alignment to atlas coordinates using cell type labels based on the BARseq datasets, and using genes using the MERFISH datasets. The BARseq datasets were clustered at three different levels [15] to reveal cell type heterogeneity, and we registered the brains using the coarsest level of cell types (52 types in the whole-coronal section dataset and 39 types in the hemi-coronal section datasets). For the MERFISH dataset, we demonstrate results for 3D alignments based on selection of 20 of the most spatially informative genes based on mutual information (see section 2.6).

### 2.3 Mapping Whole Brain to Partial Volume Censored Targets

In each technology described, we unify the molecular and tissue scales using particle (image-varifold) methods based on mathematical measures constructed from discrete “particle” Diracs, with each particle indexed by i∈I. Each particle carries a singular mass weight located in physical space ${\left(w_{i}\delta_{x_{i}}\right)}_{i\in I}$ and a conditional probability distribution ${\left(p_{i}\right)}_{i\in I}$ over the feature space [12], which may be genes or cell types or tissue types:

(2) $$\mu ≐\sum_{i\in I}w_{i}\delta_{x_{i}}⊗p_{i}.$$

For matching onto censored subvolumes, we restrict the subregion of intersection defined by the deforming atlas coordinates and the target. We introduce a set of spatially-varying weights that puncture the deforming atlas so as to select only the subvolume occupied by the target. These weights are given by the censoring function, $\alpha^{\lambda }:R^{3}\to [0,\;1]$ defined apriori in the coordinate system of the molecular/cellular target, with $\alpha_{x}^{\lambda }=1$ for all x within the support of the target and decreasing smoothly to 0 with rate of decrease controlled by λ:

(3) $$\mu^{\lambda }=\sum_{i\in I}\alpha_{x_{i}}^{\lambda }w_{i}\delta_{x_{i}}⊗p_{i}.$$

The brain mapping problem optimizes over the diffeomorphism and the support function:

(4a) $$\underset{\varphi \in Diff,\lambda }{inf}\;{\left\|\varphi \cdot \mu^{\lambda }-\mu^{\prime }\right\|}_{M}^{2}\;,$$

(4b) $$\begin{array}{c} \text{with}\;\varphi \cdot \mu^{\lambda }=\sum_{i\in I}\alpha_{\varphi \left(x_{i}\right)}^{\lambda }|D\varphi {|}_{x_{i}}w_{i}\delta_{\varphi \left(x_{i}\right)}⊗p_{i}. \end{array}$$

The physical mass of the deforming atlas, given by the product ${\left|D\varphi \right|}_{x_{i}}w_{i}$ at each location $\varphi \left(x_{i}\right)$ according to the varifold action of diffeomorphisms [10] is thus masked according to $\alpha^{\lambda }$ at that location to retain atlas regions corresponding to those with measured targets.

Importantly, these support weights allow us to distinguish subvolumes of physical space that were not measured directly in a given experiment from those that were measured but with little to no detection (e.g. gene expression) in the region. This is necessary particularly in the setting of cross-modality mapping between whole brain atlases and partial molecular targets (see Section 2.4) where diffeomorphic transformations are estimated in tandem with target feature distributions that predict both physical density of target mass per atlas region and probability distribution of that mass across features.

In the setting of whole brain coronal sections (Figure 1B,C,E,F), the support weights are computed as the sum of hyperbolic tangent functions (see Section 4.7) oriented along the rostral-caudal axis to distinguish the center portion of the CCFv3 brain (red) from the rostral and caudal most parts that were not sectioned for measurement (blue). Initial alignment between the CCFv3 and MERFISH and BARseq serial sections varied grossly in terms of rostral-caudal placement of sections (Figure 1B) and medial-lateral scale (Figure 1C), with the intersecting atlas plane largely differing in terms of initial geometry, especially in the setting of MERFISH (Figure 1H,I). Estimation of support weights facilitates accurate diffeomorphism and similtude estimation, particularly along the rostral-caudal axis in these cases where target sections are then confined to a portion of the whole brain rather than atlas and target needing to span the entire same volumes. This is seen both grossly (Figure 1E,F) and on a section-by-section basis (Figure 1L–O) where we see much stronger similarity in geometric compartments (e.g. striatum and cortical layering) following solution of the mapping problem with censoring.

In hemi-brain coronal sections, as measured with BARseq (Figure 1D,G), support weights additionally contour the irregularly-shaped central volume, with sections covering different extents (e.g. 45 – 75%) along the medial-lateral axis of the brain. The support function $\alpha_{\lambda }$ in this setting is estimated as the output of a UNET trained on the given target dataset (class 1), with dummy particles (class 0) placed along the medial boundary of each section. To represent the rostral-caudal extent of the subvolumes which are highly variable, sections of particles are placed rostral and caudal to represent the rostral-most and caudal-most sections, respectively. The final layer of the UNET feeds into a hyperbolic tangent function tanh, with the scaling parameter, λ, estimated as in the setting of whole brain coronal sections and controlling the bandwith of the transition zone along the smoothly estimated boundary (see Section 4.7 for details).

**Fig. 2:**
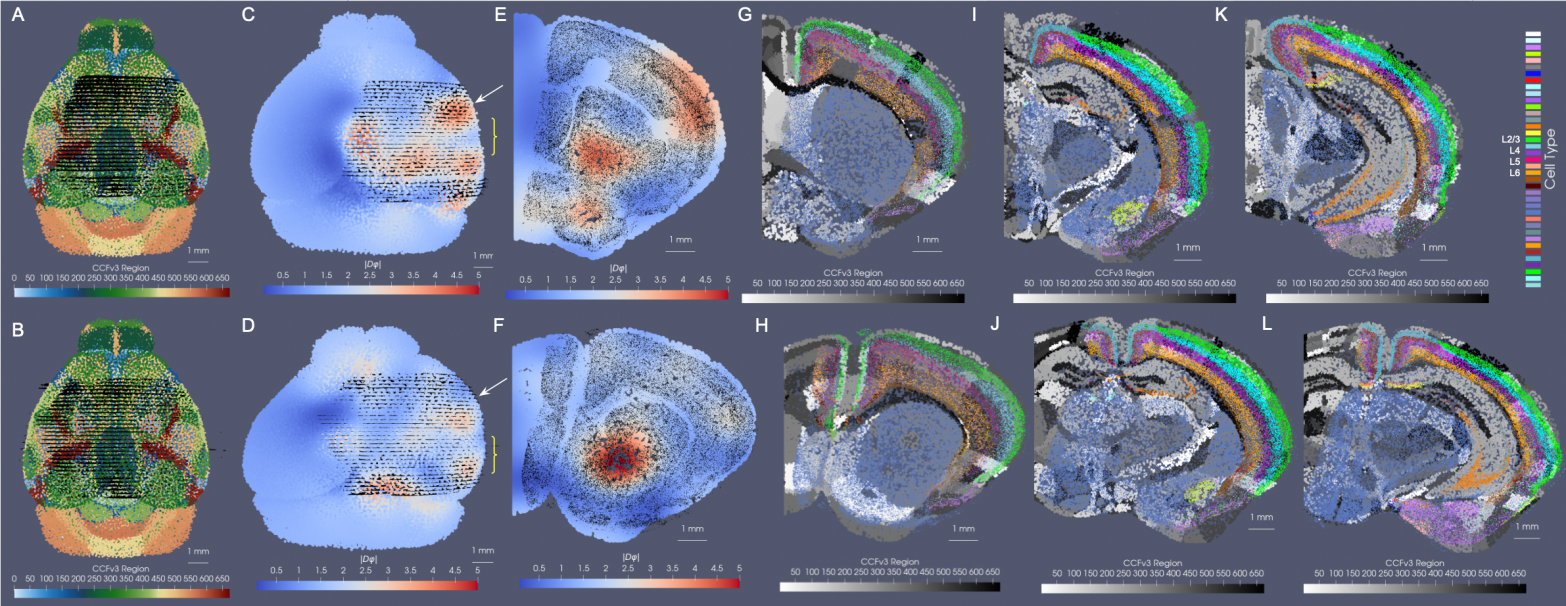
Results for mapping CCFv3 to sets of hemi-brain BARseq sections, aggregated into 200*μ*m representations for two separate mice. A,B show initial alignment of CCFv3 to BARseq sections (black dots) for mouse 1 (A) and mouse 2 (B). C,D show physically transformed CCFv3 to coordinates of target BARseq sections, with determinant of the Jacobian showing areas of expansion (red) and contraction (blue). E,F show single intersecting section of deformed CCFv3 (white arrow) overlaid with corresponding BARseq section (black dots). G-L show single intersecting section of deformed CCFv3 with ontology regions in grayscale and cell type delineations for BARseq sections overlaid in color, with G,H taken at white arrow and I-L more posteriorly (yellow bracket in C,D).

We estimated particle representations at 200*μ*m (the given spacing between parallel sections) for two stacks of ≈ 30 sections from two separate mice, which were initially aligned to within the center of the CCFv3 (Figure 2A,B). In these hemi-brain samples, hemi-coronal sections varied in span medially to the opposing hemisphere both within brain sample and across brain samples (Figure 2G–L). Per section, medial-lateral coverage spanned 50 – 70% of total coronal area. Diffeomorphic maps were estimated at multiple sub millimeter scales (1 to 0.5 mm,see Section 2.5 below) including rigid transformations at the coarsest scale to accurately align each set of hemi-sections to right-side hemispheres (Figure 2C,D). Censoring weights (e.g. Figure 1D,G) aided in generating optimal diffeomorphisms that exhibited minimal or absent deformation (e.g. with |Dφ|≈1, as shown in Figure 2C,D) within the regions of the atlas outside the support (e.g. left most side of the brain and rostral and caudal poles). Alignment between CCFv3 and each target stack of sections was observed both globally (Figure 2C,D) and on a section-wise basis with pockets of expansion interior to the tissue (Figure 2G–L) yielding alignment of boundaries around the striatum and between cortical layers according to cell type as well as the corpus callosum to regions of low cell count (few colored particles, Figure 2G–L). Importantly, alignment extended across the midline in settings of cell detections in the left hemisphere, as seen particularly in the placement of layer 2/3 cells within the CCFv3 layer 2/3 designation both on the right and left hemispheres (Figure 2H–L).

### 2.4 Crossing Modalities via Varifold Measures

Our technology for diffeomorphic mapping of the nano-scales of the transcriptome to the tissue scales of the atlas follows the framework of LDDMM [16] extended to the molecular scales as in [10, 12]. We estimate the diffeomorphism φ and similitude transformations solving the variational problem minimization of the varifold norm of (4a) via LBFGS optimization as adapted from that implemented in PyTorch.

Unique to mapping molecular/cellular datasets and tissue-scale atlases requires transformation not just in physical space but also between fundamentally distinct feature spaces defined at each respective scale (e.g. gene expression and cell distributions versus functional/anatomical delineations). Indeed, for both cellular (BARseq) and gene-based (MERFISH) datasets used throughout this work, the functional labels differ from each other and with respect to the chosen atlas (CCFv3). For each modality, we represent the functional feature with a probability law on discrete values (e.g. regions, cell types, genes). Closeness between subjects in any modality is then based on a distance (given by the varifold norm as in (4a)) between probability laws, independent of the specific feature type [10, 12].

Crossing modalities and scales as for aligning atlases to molecular/cellular targets requires us to augment the atlas with the latent variables of molecular/cellular label distributions $p={\left(p_{\ell }\right)}_{\ell \in ℒ}$ with ℒ the labels in the ontology of the atlas. We assume each region (label) in the atlas is associated to a single probability law over molecular/cellular features that holds over the entire spatial extent of the region. These probability laws are latent dimensions to be estimated at each atlas location on the feature spaces ℱ of gene or cell type. The optimization of xIV-LDDMM jointly calculates the diffeomorphism and feature laws representing the target tissue:

(5) $$\underset{\varphi \in Diff\;,p}{inf}{\left\|\varphi \cdot \mu^{p}-\mu^{\prime }\right\|}_{M}^{2}\;.$$

**Fig. 3:**
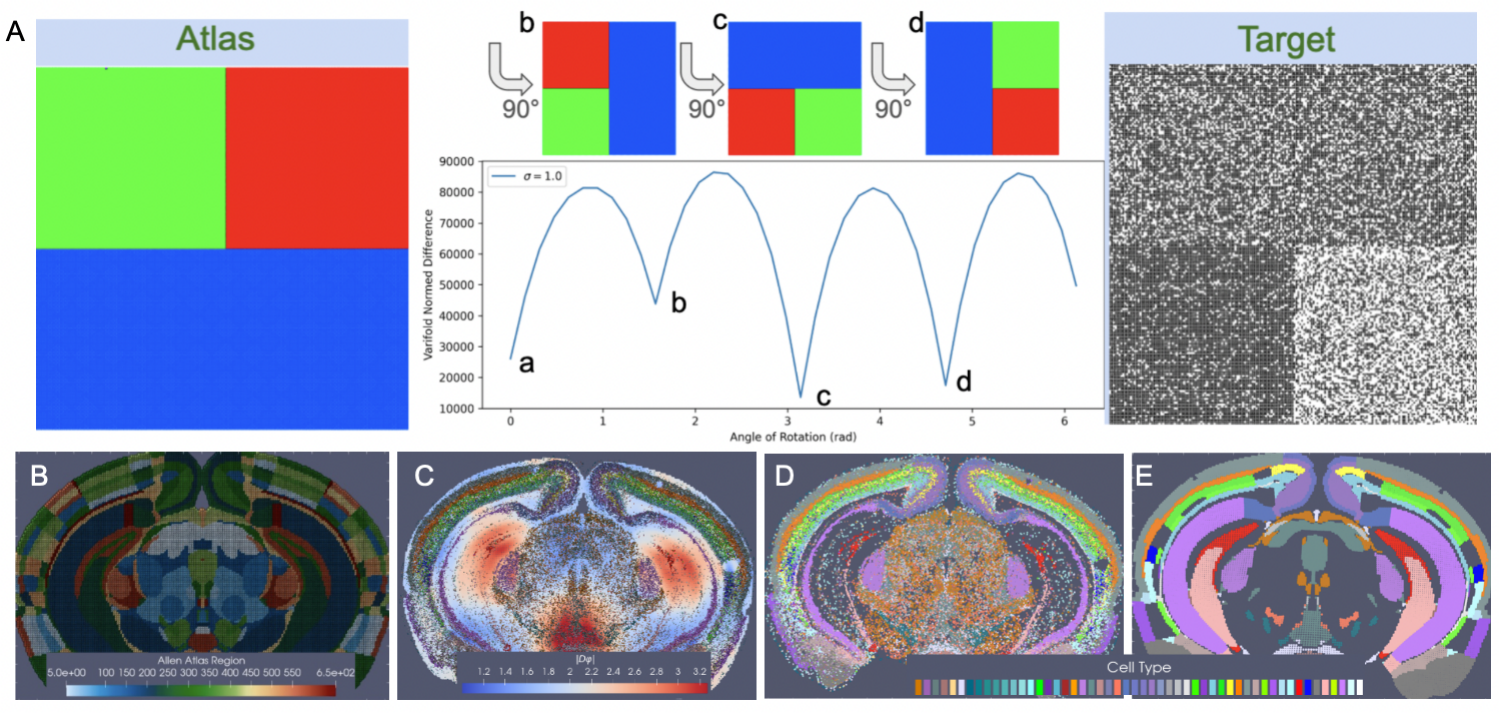
Cross-modality mapping of tissue scale atlas to molecular scale target aligning homogenous regions in space. A shows minimization in varifold norm achieved with 180 degree rotation of atlas to target when homogeneous regions (colors in atlas, black/white probability distributions) in atlas and target are aligned. B shows CCFv3 section 837/1320 (anterior to posterior). C shows transformed CCFv3 section with colors indicating determinant of the jacobian reflective of areas of expansion (red) and contraction (blue). D shows BARseq measured cells with designated cell type. E shows predicted cell type with highest probability per each transformed CCFv3 partition.

By assuming this stationarity in probability law over each atlas region, correspondence is achieved from the empirical probability laws reconstructed from the atlas and target and the relative purity of the partition, independent of the space that the data lives in. For instance, Figure 3A depicts a toy matching problem between an atlas parcelled into three regions associated to the feature space ℱ={R,G,B} and a molecular target with varying expression of two features ℱ={B,W} across the same tissue span. The correspondence is driven by the inner product between the empirical probability laws themselves, aiming to align homogeneous to homogeneous regions, not the range space of the image value of the domain that the probability law is supported on. Here, the norm distance between deforming atlas and target is thus achieved with 180 degrees of rotation in which square and rectangle boundaries parcelling space into homogeneous atlas regions and regions of homogeneous black and white feature expression are aligned.

In the setting of BARseq cell types, similar regions of homogeneity emerge, for instance, in the cortical layers and areas of the hippocampus where specific excitatory neuron subtypes predominate consistently throughout the region (Figure 3D). Correspondence between CCFv3 sections (Figure 3B) is thus achieved with diffeomorphic transformation (Figure 3C) across the section, expanding (red) and contracting (blue) areas of physical space to align these homogeneous regions while in tandem estimating per each CCFv3 region a probability law over cell types in line with what is seen in the target (Figure 3D).

### 2.5 Scale-Space Resampling

The spatial resolution of imaging in MERFISH and BARseq implies detection at the nano-scales of individual mRNA molecules each corresponding to a specific gene. The sheer complexity of what results from this sub micron imaging thus necessitates approximation based on optimal regridding for computational manipulation and downstream analysis. At any fixed complexity the particle measures are an approximation of the near infinity of potentially measured transcripts (or cells) in tissue. Unlike regular lattices used for tissue-scale imaging, the normed space our image-varifold brain objects are placed into enables the opportunity optimally to position the subspace of discrete particles being used to approximate the target at any specified complexity. We optimize resampling of the brain measure based on introducing a scale space of particle approximations, $\mu_{\sigma }$ over a series of scales, σ, which ascend in dimension as scale is refined converging to the target brain, μ:

$$\mu_{\sigma_{1}},\mu_{\sigma_{2}},\ldots ,\mu \;\text{with}\;\sigma_{1}>\sigma_{2}\ldots .$$

Particle approximation rather than being based on regridding is designed based on an optimality process enabling construction of representations at different scales (or resolutions). The natural optimality is to define the distance using the varifold norm and constructs the closest approximation in norm to μ. Each particle approximation

$$\mu_{\sigma }=\sum_{i\in I_{\sigma }}\delta_{x_{i}}⊗w_{i}p_{i},\left\{x_{i},i\in I_{\sigma }\right\},\;\text{for}\;\sigma =1,\;2,\ldots$$

is optimized over particle positions $x_{i},\;i\in I_{\sigma }$ and weighted conditional probability distributions $w_{i}p_{i}$ minimizing the normed-distance approximating the true target:

(6) $$\underset{x_{i},w_{i},p_{i},i\in I_{\sigma }}{min}{\left\|\mu_{\sigma }-\mu \right\|}_{M}^{2}.$$

Complexity is defined in terms of the number of particles representing a given brain object $\left(\propto \left|I_{\sigma }\right|\right)$. The approximations thus increase in dimension with descending scale, $\left|I_{\sigma_{1}}\right|<\left|I_{\sigma_{2}}\right|\ldots .$; in 2D thick section this scales as $O\left(1/\sigma^{2}\right)$, while for 3D, the complexity scales as $O\left(1/\sigma^{3}\right)$.

**Fig. 4:**
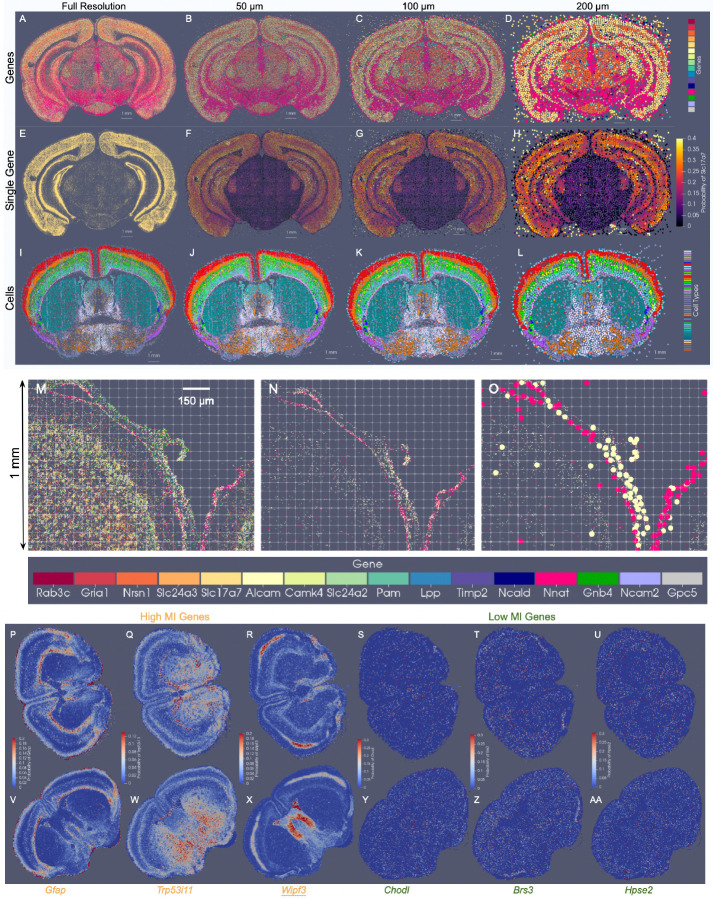
Optimized scale-space resampling based on varifold norm for genes and cell type feature spaces of BARseq and feature selection of gene space in MERFISH measured ≈ 500 genes. A shows a single section with 16 selected gene measurements totally 48 M transcripts. B-D show the gene with highest probability per particle at 50*μ*m,100*μ*m, 200*μ*m approximations with 82*k*, 21*k* and 6*k* particles, respectively. E-H show probabilities at each scale of the single *Slc17a7* gene. I-L show a single slice of BARseq cell-segmented data with 104K cells at highest resolution and at 50*μ*, 100*μ*, and 200*μ* approximations with 54*k*, 18*k* and 5*k* cells, respectively. Each cell is colored with the highest probability cell type. M shows 1mm *×* 1.25 mm window of full resolution gene detections for 16 genes (in A), and N shows detections only of Alcam and Nnat. O shows detections in N overlaid with particles whose estimated feature distributions carry the highest probability of expression for Alcam (yellow) and Nnat (pink) in optimally estimated positions along the curvilinear boundary of tissue. P-AA show relative expression of genes with high and low mutual information (MI) scores across two MERFISH tissue sections (P-U, V-AA) at coronal levels *Z* = 385 and *Z* = 485 in CCFv3 coordinates, respectively. Genes with highest mutual information scores (P-R, V-X) are *Gfap*, *Trp53i11*, *Wipf3*) and lowest (S-U, Y-AA) are *Chodl*, *Brs3*, *Hpse2*.

The generalizability of the image varifold representation, as described, extends to this setting where we can estimate cascades of optimized scale-space representations across scales ($\sigma_{3}=50,\;\sigma_{2}=100,\;\sigma_{1}=200\;\mu m$)and for different feature spaces of different (e.g. gene and cell-types) (Figure 4A–L). Notably, dominant genes (Figure 4B–D) and dominant cell types (Figure 4J–L) estimated per particle reflect the spatial patterns of gene expression and cell distribution in the full resolution dataset (Figure 4A,I). Furthermore, particles in approximations carry not just a single most likely feature, but distributions over feature types reflective of larger and larger neighborhoods (Figure 4F–H). This enables the interrogation of the spatial probability distribution of discrete features, such as single genes. In a single BARseq coronal section, for instance, areas of high probability of expression of specific genes (e.g. *Slc17a7*) accurately reflect high density areas of expression of said gene at the highest resolution (Figure 4E), consistent with the accuracy of our scale-space resampling, with those areas of highest expression being those of the hippocampus and both the outer and innermost layers of cortex compared with the middle layers (Figure 4E–H).

Importantly, particle measures in the reduced set are initialized with uniform distributions over features and with total mass given by the σ-neighborhood of highest scale (full resolution) measures. Without restriction to a regularized grid, spatial positions of particles move to optimally align the feature distributions based on the varifold norm, thereby reflecting what is more likely a curvilinear geometry of the tissue elements (Figure 4). Particles approximated along the central sulcus, for instance, (Figure 4C) begin with uniform distributions and are further differentiated according to feature distribution with particular genes predominating (probability of Nnat in the pink dots is 0.37 +/− 0.15 and probability of Alcam in yellow dots is 0.33 +/− 0.15), as observed at high resolution (Figure 4A,B). As we are approximating at a scale of 50 um, particles move on average 9.7 um +/− 6.5 um, to adjust to the specific geometry/curvature of the tissue and distribution of the underlying genes.

We have compared the optimization based scale-space resampling to alternative schemes of regridding and clustering (Supplementary Table 1). For K-means clustering, we follow Lloyd’s algorithm [17], with number of clusters, K=20000, and positions initialized to those of a randomly selected subset of K particles from our starting set of ≈ 5 million. The reduced set of particle measures carry spatial positions as the estimated centroids and weighted conditional probability distributions comprised of the sum of masses per feature of the high resolution particles assigned to the corresponding cluster. For grid resampling, we follow the scheme in [9] for crossing scales, where particle measures are redistributed in space according to a given spatial kernel.

Methods such as K-means place the particles on a regularized grid so as to minimize the physical distance only to the high resolution particles, rather than account for proximity in both physical and feature space. This strategy thus generates particle measures that carry a much greater distance with respect to the varifold norm to the high resolution target than our defined scheme here (Supplementary Table 1). This underscores the relevance of our varifold norm approximation method for achieving data reduction in the context of mapping tissue-scale atlases to molecular and cellular-scale targets with xIV-LDDMM.

### 2.6 Feature Selection via Mutual Information

The complexity of transcriptomic measurements not only requires reduction in physical space, as captured by scale-space resampling, but often also in feature space via a feature selection mechanism. A common selection mechanism is to consider those genes that are most “spatially variable” [18] or “differentially expressed” [19] under the assumption that expression pattern thereby varies per biologically different regions of tissue. This is particularly relevant, here, in the context of mapping spatial transcriptomics datasets to atlases where we aim to estimate distributions over genes for each region in our atlas that we assume is homogeneous within the region. Various methods have been described for identifying which genes in a spatial transcriptomics dataset are spatially varying including hierarchical classification [20] and modelling based on covariance statistics and Gaussian process models [18], [21]. Since particle methods, as used here, imply sparsity in number, methods based on spatial Poisson and point-process models, as introduced in [10], underscore the fundamental roles of Kullback Lieber (KL) divergence between empirical distributions derived from the field to score hypothesis reliability. KL divergence has also been proposed in [19] for selecting genes with differential expression across cells distributed in space.

Here, we introduce information theoretic methods based on mutual information scoring which assess the differential expression of genes in space in a cell-independent manner, scoring highly genes that are most spatially varying (see Section 4.5). For this we define the space of random variables denoted X reflecting the vertical and horizontal discontinuities associated to partition boundaries (Supplementary Figure 1), and the family of features G capturing statistics based on mRNA expression (or cell-type). Our mutual information selection procedure is greedy in ordering the conditional entropy of every gene g∈ℱ in discrimination of the up-down/left-right boundaries:

H(X∣G(1))>H(X∣G(2))>H(X∣G(3))……

Our greedy mutual information procedure chooses the first n genes implying that if the genes are independent then we have the monotonic ordering of the mutual information,

I(X;G(1))<I(X;G(1),G(2))….

For mapping the Allen CCFv3 [4] to serial sections of MERFISH data [2, 3], we ordered the 500 genes measured by computed mutual information score and selected the subset of 20 with the highest scores to comprise a reduced feature set. Scores ranged from ≈ 470000 – 200000 roughly following a single mode distribution. In line with expectations, 75% of the genes comprising those with scores in the bottom 25% of the total 500 genes were decoy genes (e.g. ‘BLANK’) without biological meaning but used as controls for assuring the quality of the dataset. Non-decoy genes exhibiting scores in the bottom 2% of the entire gene set were *Chodl*, *Brs3*, and *Hpse2*, whose expression patterns carried high local variance, but with stationarity observed over entire tissue sections, from region to region (Figure 4S–U,Y–AA). In contrast, genes with the highest mutual information scores included *Gfap*, *Trp53i11*, and *Wipf3*, where certain areas of tissue exhibited high local density in contrast to others (Figure 4P–R,V–X). For such genes, the observed delineations with respect to expression level equated to many complementary delineations given in the CCFv3 ontology, such as that of the corpus callosum exhibiting high levels of *Gfap* expression (Figure 4P) or the lateral septal nucleus exhibiting high levels of *Wipf3*, (Figure 4R), thereby underscoring the relevance in these genes’ expression patterns as target feature measures for aligning to the regions in the CCFv3.

### 2.7 Accuracy of 3D Mapping of BARseq to Allen CCFv3 Atlas Based on Cell Types

**Fig. 5:**
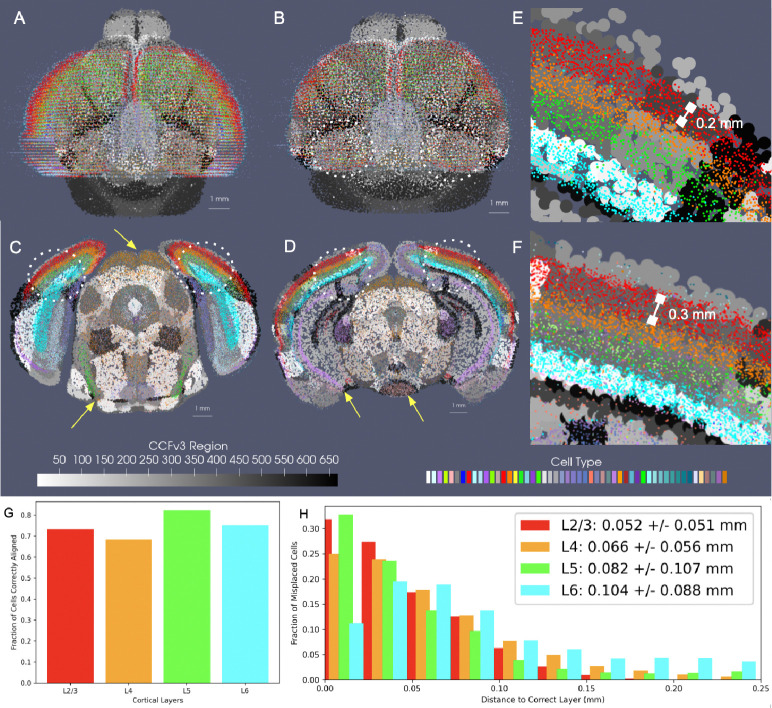
Accuracy of mapping between CCFv3 and whole-brain BARseq sections (*n* = 40), carrying cells of ≈ 50 different subtypes, with each dataset approximated at 200 *μ*m. A shows initial alignment of CCFv3 (grayscale) to whole-brain BARseq sections (color). B shows alignment in BARseq coordinates following diffeomorphic transformation of CCFv3. C,D show intersecting planes of transformed CCFv3 with two BARseq full resolution cellular sections (*n* = 80–100k cells per section). Yellow arrows point to areas of correct alignment between designated BARseq cell types and regional CCFv3 delineations. E and F show zoomed in region of of lateral visual area and primary somatosensory cortex (dotted circles) in tissue sections in C and D with individual cells overlaying corresponding layers in CCFv3 grayscale delineations. Areas of the dentate gyrus and hippocampus show some misalignment in dorsal-ventral placement. G shows percentages of correctly aligned cells across 10 sections bilaterally in the area of the primary somatosensory cortex and lateral visual area to corresponding CCFv3 cortical layer designation. Total cells mapped per layer are 23660 (L2/3), 19796 (L4), 11194 (L5), 22928 (L6). H shows the distance of incorrectly aligned cells to the correct corresponding layer designation.

We evaluated the efficacy of our joint optimization scheme through association of delineated cell types in BARseq whole brain sections to respective cortical layers delineated in the CCFv3. The cell types were clustered with an algorithm similar to Louvain-clustering to achieve a resolution analogous to the “subclass” defined in RNA-seq studies [20]. These included both inhibitory and excitatory cell types specific to layers 2, 3, 4, 5, and 6 (Figure 5). Global alignment of the CCFv3 to BARseq stack was observed along the rostral-caudal axis and medial-lateral boundaries (Figure 5B) as a result of estimating both similitude transformations and diffeomorphism to correct differences in scale and tissue geometry as seen in Figure 5A. Within the tissue plane of individual sections (Figure 5C–F), we observed alignment between regions predominated by single BARseq cell types and corresponding regional CCFv3 delineations both ventrally and dorsally. For instance, subtypes of gabaergic neurons predominant in areas of the superior colliculus (yellow arrow at dorsal orange, C), lateral lemniscus (yellow arrow at ventral green, C), and medial mammillary nucleus (yellow arrow at ventral peach, D) generated contours of these regions that aligned to the corresponding delineations of the CCFv3. Likewise, designations of pyramidal cells in areas of the hippocampus (e.g. CA1, yellow arrow at purple band, D) localize closer to the lateral/deeper aspects of the CCFv3 designated regions, as we expect anatomically.

We further quantified the accuracy of alignment across the cortical layers within the neighborhood of the primary somatosensory cortex and lateral visual area (dotted white circles) by assessing the fraction of full resolution cells aligning to the correct corresponding layer according to cell type within these regions. Cortical cells were selected bilaterally across ≈ 10 sections intersecting these regions. Percentages of correctly aligned cells ranged from ≈ 70 – 80% across the four layer designations with misaligned cells falling on average ≈ 50*μ*m from the correct layer (Figure 5G,H) and thereby reflecting misalignment to adjacent layers only, with layers ranging in thickness from ≈ 100*μ*m to 300*μ*m (Figure 5E,F). Notably, these misalignment percentages reflect both errors in alignment as well as variation in cell typing, naming cells physically within certain layers with type corresponding to a different layer. For comparison, we computed similar percentages of cells accurately matched to CCFv3 layer following manual alignment of BARseq hemi-brain sections (as seen in Figure 2) to the CCFv3 (see Supplementary Table 3). Across 7 hemi-brain samples, percentages of correctly aligned cells across 4 layer designations were lower on average with manual alignment in layers 4, 5,and 6 compared to those achieved with our alignment procedure in this whole brain (0.79 ± 0.04, 0.57 ± 0.04, 0.74 ± 0.02, and 0.70 ± 0.02 versus 0.73, 0.68, 0.82, 0.75).

### 2.8 3D Mapping of MERFISH to Allen CCFv3 Based on Genes

As illustrated in the setting of cell-typed sections (Section 2.7), there exist certain 1:1 relations between organization of cell types and demarcated tissue regions, as defined in the CCFv3 ontology (e.g. in cortical layers), that serve to anchor alignment between these scales of data and that can be harnessed directly to assess accuracy of this alignment. At the sub cellular scale, however, we observe and expect inherently more variation over space in terms of gene expression with cell typing typically used to capture stable co-expression relationships among genes. As such, gene expression over physical space organizes into different sizes and shapes of homogenous regions where some boundaries are preserved across tissue, cellular, and sub cellular scales (e.g. striatal boundaries) and others differ (e.g. medial to lateral and dorsal to ventral within the cortical layers). This inherently increases the difficulty in achieving alignment between these subcellular datasets and tissue-scale atlases that exist at even further discordant scales.

**Fig. 6:**
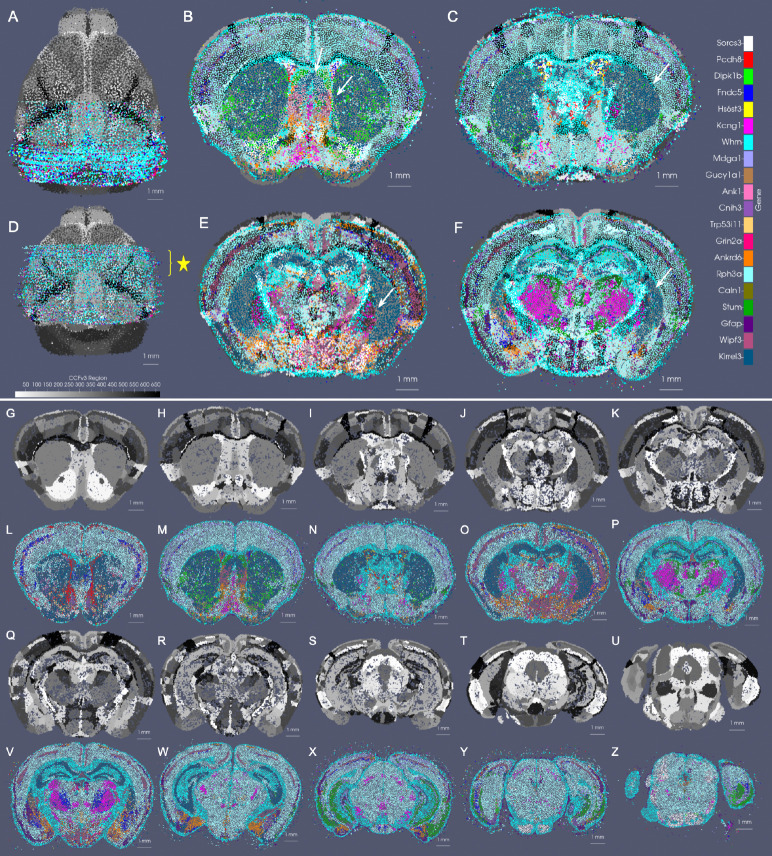
CCFv3 Alignment to MERFISH reads of 20 selected spatially variable genes. A shows initial alignment of CCFv3 to stack of 60 sections, each sampled at 200 *μ*m resolution. D shows final alignment of CCFv3 to stack of MERFISH sections following rigid and diffeomorphic transformation. B-F show in-plane alignment of intersecting CCFv3 to 4 selected MERFISH sections in the anterior half of the mouse brain (spaced 500 microns apart, yellow star in D). Alignment between foreground-background tissue boundaries is seen particularly along the ventral and lateral edges. White arrows show matching contours of outer and inner boundaries of the CCF striatum to areas predominantly expressing Kirrel3. White arrows also illustrate alignment within the area of the lateral septal nucleus (B). G-Z show sections every 0.5 mm (L-P,V-Z) with corresponding intersection (G-K,Q-U) through deformed atlas following alignment, as in (D).

To demonstrate the amenability of our mapping strategy to bridging the tissue to the subcellular scales, we mapped the CCFv3 to a set of 60 MERFISH sections exhibiting expression of 20 of the 500 measured genes we selected as spatially variable (see Section 2.6) according to mutual information score (Figure 6). Alignment along the rostral-caudal axis, as facilitated by our incorporation of censoring to focus accuracy of matching within the MERFISH tissue support (see Section 2.3) was observed globally (Figure 6D) and on a section-by-section basis with areas of the striatum and hippocampus appearing in similar proportions and shapes in both the CCFv3 and gene space of MERFISH (Figure 6G–Z). Medial-lateral and inferior-superior alignment was also observed in the matching of cortical versus striatal boundaries that appeared in both CCFv3 ontological delineations and the gene space of MERFISH (Figure 6B–F). Hence, our mapping strategy is amenable to these different granulations of parcellation in succeeding to situate both interior (e.g. striatal) and exterior (foreground/background) geometric delineations to one another across tissue and cellular architecture (Figure 5) as well as ultimately tissue to gene expression architecture (Figure 6).

### 2.9 Computational Complexity of 3D Particle Codes

We have examined the effects on runtime and estimated diffeomorphism in mapping a single consistent tissue-scale atlas to particle representations of varying size and feature spaces of the stack of 40 whole brain coronal BARseq sections. Particle representations at scales of 200, 100, and 50*μ*m corresponding to discrete particle numbers of *o*(5000), *o*(20,000), *o*(50,000) per single section, respectively, were estimated with optimized scale-space resampling (see Section 2.5) for the BARseq sections over the entire feature space of 52 cell types (Supplementary Figure 2D–F).

Supplementary Table 2 shows the complexity and corresponding memory requirements to store the particle representations. Runtime is computed as total time to run 150 iterations of the optimization scheme for estimating the geometric transformation and feature distributions. Memory requirements are tallied as the amount of storage per target dataset given as *.pt* file. Resolution, # particles, and # features are all given for the target image varifold. Runtime measures were computed on an NVIDIA RTX A5000 GPU. Feature distributions and diffeomorphisms, parameterized by a set of initial momenta numbering *o*(60000) for the number of particles in the 200 *μ*m CCFv3 representation, were estimated to transform the CCFv3 atlas to each of the target representations via minimization of (11), with $K_{\sigma }$ chosen as the sum of two gaussian kernels at 150 and 750*μ*m.

Both memory requirements and runtime increased, particularly in the setting of utilizing a 50*μ*m aggregated representation compared with one at 200*μ*m (Supplementary Table 2), thereby supporting the use for reductionist mechanisms to treat the high initial complexity of this data. Importantly, however, we observed no difference in estimated diffeomorphic transformation, as evidenced by the determinant of the Jacobian depicting the distribution and magnitude of expansion and contraction (Supplementary Figure 2G–I). The equivalence in mapping results across the experiments run with different scales of target approximation was also reiterated in the observed global alignment and slice-wise alignment of BARseq particle measures (black) overlaying corresponding areas of CCFv3 delineated tissue (Supplementary Figure 2A–C and J–L). Alignment of the cortical layers was visually estimated in each case to be within 25 – 50*μ*m, as evidenced by layer 1 of the CCFv3 falling outside the outermost cell type of layer 2/3, and layer 6 dorsal to the corpus callosum (Supplementary Figure 2M–O). Notably, the equivalence in estimated mappings across these three scales of particle representations supports the integrity with which the scale-space resampling scheme (Section 2.5) holds to the high resolution data at each scale.

## 3 Discussion

This resource paper describes a set of technologies that enable cross-modality mapping across tissue, cellular, and molecular scale data in settings often of peta-scale complexity that arise particularly in 3D. We have specifically introduced modules for (i) matching partial to whole tissue volumes with censoring, (ii) cross-modality mapping based on an image-varifold measure norm, (iii) scale-space optimization for multi-scale resampling, and (iv) mutual information-based feature selection. Importantly, while these technologies can be employed independently for data reduction and analysis, they are rooted in the image-varifold (particle measure) representation to facilitate integration amongst them. Collectively, these technologies thus afford efficient representations of peta-scale data and mapping with increasing refinement across millimeter to nanometer scales.

While we have focused, here, on the complexity challenges posed even in the setting of mouse brains, we would expect this complexity only to increase in the setting of larger primate and ultimately human brains, where we already see similarities and need for the censoring and reduction schemes we introduced here. In brain imaging problems, for instance, subvolumes and/or sparse data sets are almost always collected, with a focus on particular regions involved in particular diseases. For instance, in Alzheimer’s disease, the medial temporal lobe [22] is often the region of study, whereas in Huntington’s disease, the subcortical structures[23] are the focus. Spatial transcriptomics technologies have similarly limited capture to within subregions, often to optimize resolution within these regions at the expense of further breath across tissue. In mapping problems, while whole volume objects could be subsectioned prior to mapping according to known subvolume covered by a molecular or cellular dataset, this often is not known apriori and prevents easy integration of multiple molecular and cellular datasets into a single unified coordinate framework that may only partially overlap with regard to subvolumes measured.

In differential geometry, the emphasis is to represent the whole via a collection of local charts which form a complete covering; the atlas is thus a collection of local charts. This implies the diffeomorphic mapping problem is surjective, as we have examined in [24]. Here, we provided a general strategy for solving the “chart problem”, which significantly expands the partial matching approaches in the setting of medical images modeled in [25], by appending the inner-product with a censoring function (4a) (Section 2.3). The scheme for partial matching or censoring the full tissue-scale atlas to molecular and cellular datasets spanning only subvolumes uses a masking function that punctures the corresponding subvolume of the target within our deforming atlas. This effectively restricts the matching criteria to the subvolume within which molecular/cellular data has been detected to drive both geometric transformation and feature distributions fundamentally to reflect the biological boundaries in the data present and not those artificially induced through only partial captures of tissue volume.

The optimization based multi-scale resampling we propose benefits from the fact that we place the brains into a Hilbert space, which enables optimized approximations at any scale, i.e. complexity. Furthermore, while the scheme parallels traditional clustering schemes (e.g. K-means), these clustering schemes are often limited to defining distance only with regard to “location” in either physical or feature space, but not both, as is innately captured by our image-varifold normed space. Rasterization (as in [26]) and resampling onto regular lattices, for instance, as means of aggregation focus exclusively on proximity in physical space. Clustering based exclusively on feature type (e.g. gene) has also been described, with particular support of clustering schemes not assuming particular shapes or sizes to clusters (e.g. Louvain cluster) [27]. While some efforts to combine clustering along both axes have achieved this by applying tactics independently and sequentially, many have focused on jointly estimating a single lower-dimensional representation that aptly captures the structure of the data along both physical and feature axes [28–31]. Indeed, with many as generative schemes, such as SpatialPCA and BayesSpace, these joint approaches have been used not only to reduce data to set of lower dimensions, but to use such dimensions to generate higher (subspot) resolution of original datasets [29, 30]. Here, we demonstrated that for a fixed particle grid, the distances of the measure approximation to the original varifold is significantly less than many of the other regridding methods including K-means and nearest-neighbor. Furthermore, we showed in our optimization scheme that particle measures not only change position to approximate the ideal, often curvilinear geometry of the tissue, but they also change dramatically the functional feature to more appropriately match the transcriptome or cellular target.

Finally, we also have defined an information theoretic greedy procedure for choosing a subset of functional features from ℱ according to the mutual information gain (Section 2.6). The goal is to identify features with highest spatial variability and therefore, most likely to be informative in denoting boundaries to which tissue-scale atlas regions might align.

Here, we have demonstrated the efficacy of xIV-LDDMM, shown efficacious previously in a 2D setting [12], but now coupled to these schemes of data reduction and partial matching (censoring) for mapping a complete 3D CCFv3 to molecular (gene-based) and cellular transcriptomics datasets from different technologies (MERFISH and BARseq) and with varying scopes of capture from whole brain to hemi-brain sections. Strengths of this approach include the generalizability of the image-varifold representation to data detected within different sampling schemes (e.g. spot-technologies versus single-cell measurement schemes) and the modeling of 2D stacks as 3D objects for mapping to whole brain 3D coordinate systems. In contrast, many current schemes [32, 33] treat sections independently, which necessarily requires apriori selection of 3D atlas section to which to align each molecular/cellular section, which is challenging in typical settings in which tissue is not necessarily sectioned perpendicular to a given axis.

While the focus of this manuscript has been on the description and evaluation of the data reduction and mapping schemes associated to xIV-LDDMM, one avenue of future work includes further exhibition of the generalizability of the approach to mapping molecular and cellular datasets from additional technologies (e.g. SlideSeq, synapse imaging [8]). Furthermore, as described in [12], estimation of a diffeomorphic transformation taking tissue-scale atlas to molecular or cellular-scale target affords the pulling back of each target into the atlas common coordinate framework via the inverse transformation. This consequently enables integration of measures across replicates and technologies for comparison of feature distributions across them.

A second avenue of future work is in selection and expansion of the feature spaces modeled per image-varifold object. First, we have described a greedy feature selection procedure looking at genes (or cell types) independently to select an informative subset. It is natural to study such selection in the context of non-independence, examining pairs and triples, with many cell types or genes distributed similarly with functional associations. This is something we are currently pursuing. Second, though each dataset here was represented as an image varifold over a single physical and feature space, the coupled modeling of different feature spaces (e.g. genes and cell types) over which a single image varifold is defined naturally addresses the incorporation becoming prevalent at the stage of data collection. For instance, cell-based datasets, such as the BARseq example analyzed here, often carry both gene and cell-level information based on integration of raw measurements of mRNA with segmentation and clustering schemes (e.g. histological stains, clustering). The image-varifold representation lends itself to associating to physical spatial measures not just single feature measures but potentially feature measures over differing feature spaces (e.g. genes, cell types) that could simultaneously be used for mapping between tissue-scale atlases and these molecular/cellular scale datasets.

Finally, a significant challenge of the xIV-LDDMM algorithm is the peta scale nature of the quadratic computation. At its core, each particle and feature are compared to each other one in the inner-product Hilbert space of varifold norms. This computational complexity is illustrated by the run times on the order of 24 hours for computing single mappings (see Table 2). Our strategy thus far has been to exploit the parallelism of GPUs. The multi-scale resampling scheme described in Section 2.5 naturally lends itself to the multi-scale mapping scheme initially introduced in [9], in which successive refinements can be made at coarse to fine scales of estimated transformations. In a multi-scale approach, we aim to estimate transformations first at a coarser scale with fewer particles needed to capture our atlas and target objects. Successive addition of finer scale representations for refining these coarse scale mappings can then be achieved with likely fewer iterations, reducing the overall computational load these mapping schemes encompass. We are currently working to adapt the scheme described here to a multi-scale setting for improved efficiency in the future and with the prospect also of accommodating different feature spaces over which particle representations might be defined at each scale.

## 4 Methods

### 4.1 The Brain Mapping Model for Molecular Scales

Our molecular representations at the micron and submicron scales are represented mathematically as a set of discrete “particle” Diracs indexed by i∈I, each carrying a singular mass weight located in physical space at ${\left(w_{i}\delta_{x_{i}}\right)}_{i\in I}$ and a conditional probability distribution ${\left(p_{i}\right)}_{i\in I}$ over a feature space [12] $\mu ≐\sum_{i\in I}w_{i}\delta_{x_{i}}⊗p_{i}$. The norm is associated to the brains modeled in the Hilbert space with inner product giving

(7) $$\langle \mu ,\mu \rangle_{M}≐\sum_{i,j\in I}w_{i}w_{j}K_{\sigma }\left(x_{i},x_{j}\right)\sum_{f,g\in ℱ}K_{F}\left(f,g\right)p_{i}\left(f\right)p_{j}\left(g\right).$$

The normed-space takes the brains as a reproducing kernel Hilbert space [9, 10, 12] with space kernel, $K_{\sigma }$, typically a Gaussian or other forms of Matern kernels, and $K_{F}$ often used as an indicator function for gene or cell labels or a Euclidean inner product for real-valued features [10]. Thompson’s brain mapping [11] is defined through the space of transformations defined as group actions by diffeomorphisms φ∈Diff,

(8) $$\varphi \cdot \mu ≐\sum_{i\in I}|D\varphi {|}_{x_{i}}w_{i}\delta_{\varphi \left(x_{i}\right)}⊗p_{i}$$

The normed distance between deforming atlas and target is then:

(9) $${\left\|\varphi \cdot \mu -\mu^{\prime }\right\|}_{M}^{2}=\langle \varphi \cdot \mu ,\varphi \cdot \mu \rangle_{M}-2{\left\langle \varphi \cdot \mu ,\mu^{\prime }\right\rangle }_{M}+{\left\langle \mu^{\prime },\mu^{\prime }\right\rangle }_{M}$$

with the brain mapping problem transforming one brain to the other by minimizing

(10) $$\underset{\varphi \in Diff}{inf}\;{\left\|\varphi \cdot \mu -\mu^{\prime }\right\|}_{M}^{2}\;.$$

Flows are introduced to generate the diffeomorphisms giving the variational problem.

#### Variational Problem 1.


(11) $$\begin{array}{l} \underset{v\in L^{2}([0,1],V)}{inf}\frac{1}{2}\int_{0}^{1}\;{\left\|v_{t}\right\|}_{V}^{2}dt+{\left\|\varphi_{1}^{v}\cdot \mu -\mu^{\prime }\right\|}_{M}^{2}\\ \;with\;\varphi_{t}^{v}=\int_{0}^{t}\;v_{s}\circ \varphi_{s}^{v}ds+Id,t\in [0,\;1]. \end{array}$$

### 4.2 Mapping Across Scales to Atlases

Tissue scale atlases have an associated partitioning scheme with individual regions indexed by ℓ∈ℒ, (e.g. |ℒ|<1000 for CCFv3), but do not generally carry the functional features of genes and cell-types. For diffeomorphic mapping of tissue-scale atlases onto the feature space of molecular/cellular scale targets, we associate the latent gene/cell-type feature laws to the atlas $\mu_{A}^{p}$. We index by partition class the non-normalized laws on features, $\left(p_{\ell }\left(f\right),f\in ℱ\right),\;\ell \in ℒ$, with normalized probability laws given as ${\overset{‾}{p}}_{\ell }=\frac{p_{\ell }}{\sum_{f\in ℱ}p_{\ell }(f)}$. Associated to each point in the atlas, per its representation as an image-varifold object, is the probability of a partition class, $\pi_{i}(\ell )$, ℓ∈ℒ, which then dictates the molecular/cellular feature law to which it is associated, giving the atlas with estimated gene/cell-type features as:

$$\mu_{A}^{p}=\sum_{i\in I}w_{i}\delta_{x_{i}}⊗\sum_{\ell \in ℒ}\pi_{i}\left(\ell \right)p_{\ell }.$$

Diffeomorphic transformation then acts on this object to give:

(12) $$\varphi \cdot \mu_{A}^{p}≐\sum_{i\in I}|D\varphi {|}_{x_{i}}w_{i}\delta_{\varphi \left(x_{i}\right)}⊗\sum_{\ell \in ℒ}\pi_{i}(\ell )p_{\ell }$$

which is aligned to molecular/cellular target. Both diffeomorphism and feature distributions, φ and ${\left(p_{\ell }\right)}_{\ell \in ℒ}$ are estimated through minimization of the varifold normed distance between transformed atlas and target:

(13) $${\left\|\varphi \cdot \mu_{A}^{p}-\mu^{\prime }\right\|}_{M}^{2}={\left\langle \varphi \cdot \mu_{A}^{p},\varphi \cdot \mu_{A}^{p}\right\rangle }_{M}-2{\left\langle \varphi \cdot \mu_{A}^{p},\mu^{\prime }\right\rangle }_{M}+{\left\langle \mu^{\prime },\mu^{\prime }\right\rangle }_{M}$$

which gives the variational problem associated to our cross-modality mapping scheme (xIV-LDDMM).

#### Variational Problem 2.


(14) infv∈L2([0,1],V),pℓ,l∈L12∫01vtV2dt+∑ℓ∈ℒJKLpℓ+φ1v⋅μAp-μTM2φtv=∫0tvs∘φsvds+Id,t∈[0,1].

Here note we have added a “prior” term $J_{KL}\left(p_{\ell }\right)$ weighting the probability laws towards the uniform distribution with KL divergence (see Section 4.4).

### 4.3 Rigid Alignment of Tissue Sections

Apriori, individual sections in a given stack from a single specimen may not be aligned to each other. Hence, the 2D sections are first rigidly aligned to one another, independent of an atlas, as in [34] to account for differences in orientations that result from the imaging process. To solve for the low-dimensional rigid motions to bring each section into alignment with each other, we directly minimize with LBFGS (with implementation adapted from that in PyTorch) the varifold norms between each section associating to them the rigid motions. Each section is centered around its origin. For a stack of N sections, we fix first (n=1) and last (n=N) sections in a given stack (e.g. rostral-most and caudal-most) and then estimate for all intermediate sections a rotation, and 2D translation $\left(R_{\theta },\tau \right)$ minimizing the pairwise varifold normed distance between adjacent sections in the stack:

(15) $$\underset{\left(\theta_{n},\tau_{n}\right),2\leq n\leq N-1}{min}\;{\left\|\mu_{1}-\mu_{2}^{\theta_{2},\tau_{2}}\right\|}_{M}^{2}+{\left\|\mu_{N-1}^{\theta_{N-1},\tau_{N-1}}-\mu_{N}\right\|}_{M}^{2}+\sum_{n=2}^{N-2}{\left\|\mu_{n}^{\theta_{n},\tau_{n}}-\mu_{n+1}^{\theta_{n+1},\tau_{n+1}}\right\|}_{M}^{2}\;\;\text{with}\;\mu_{n}^{\theta_{n},\tau_{n}}≐\sum_{i\in I_{n}}w_{i}\delta_{R_{\theta_{n}}x_{i}+\tau_{n}}⊗p_{i}.$$

The varifold normed distance ${\left\|\;\cdot \;\right\|}_{M}^{2}$ is defined as in (7), with $K_{\sigma }$ a gaussian kernel and $K_{F}$ the identity kernel.

### 4.4 LDDMM Mapping Tissue-scale Atlas to Molecular/Cellular-scale Targets

To solve for the high-dimensional diffeomorphisms for the variational problems of (1) and (2), we directly minimize with LBFGS the varifold norms using geodesic shooting to generate the flow of diffeomorphisms onto the target as measured with the varifold norms. Previously we have described alternating optimization schemes separating the unknown laws ${\left(p_{\ell }\right)}_{\ell \in ℒ}$ from the diffeomorphism using quadratic programming [10, 12]. Here we use a single optimization scheme introducing regularization with constraints for $p_{\ell }>0$.

As introduced in [9, 35], in geodesic shooting we model the velocity v as the control of the dynamical system which is parameterized in the momentum variables of the system $\left(\rho_{i}^{x},\rho_{i}^{w}\right)$ representing “space” momentum and “mass” momentum, respectively:

(16) $$v\left(x\right)≐\sum_{i\in I}k_{\sigma }\left(x,x_{i}\right)\rho_{i}^{x}+\frac{x\;-\;x_{i}}{\sigma^{2}}w_{i}\rho_{i}^{w}k_{\sigma }\left(x,x_{i}\right).$$

We define the Gaussian kernel $k_{\sigma }(x,y)=exp(-\frac{{\left\|x-y\right\|}_{2}^{2}}{2\sigma^{2}})$ with scale bandwith σ. The kernel $k_{\sigma }$ determines the spatial scale of the system of solutions defining the Green’s kernel of the RKHS with norm ${\left\|\;\cdot \;\right\|}_{V}^{2}$ controlling the smoothness of the flows of diffeomorphisms [36].

In geodesic shooting, velocity of the system controls the flow and is reparameterized by only the initial value of momentum at time $t=0,\;\left(\rho_{0}^{x},\rho_{0}^{w}\right)$ which determines the entire geodesic path of the atlas to target. The Hamiltonian of the system determines the geodesic equations for the flow of the state $q_{it}=\left(x_{it},w_{it}\right),\;t\in [0,\;1]$ and the momentum $\left(\rho_{it}^{x},\rho_{it}^{w},t\in [0,\;1]\right)$ whose dynamics are given by the Hamiltonian of our system:

(17) $$H\left(q,\rho ,v\right)=\sum_{i\in I}\rho_{i}^{x},v\left(x_{i}\right)+\rho_{i}^{w},div\left(v\left(x_{i}\right)\right)w_{i}-\frac{1}{2}{∥v∥}_{V}^{2}$$

where $x,\;w,\;\rho^{x},\;\rho^{w}$ denote the set of variables per particle i∈I.


(18) $${\dot{q}}_{i}=\nabla_{\rho_{i}}H=\left\{\begin{array}{l} \dot{x_{i}}=v\left(x_{i}\right)\\ {\dot{w}}_{i}=div\left(v\left(x_{i}\right)\right)w_{i} \end{array}\;.\right.$$

(19) $${\dot{\rho }}_{i}=-\nabla_{q_{i}}H.$$

As each particle measure is associated to a pair of momenta $\left(\rho_{i}^{x},\rho_{i}^{w}\right)$, the total parameter set is o(|I|), parameterizing the estimated control.

In the joint optimization scheme, we additionally need to constrain our estimates of the latent gene/cell-type feature laws. To constrain these $p_{\ell }>0$, we use a penalty defined by KL divergence of $\bar{p_{\ell }}$ (the normalized probability law) to a uniform distribution $\frac{1}{\left|ℱ\right|},\;\forall f\in ℱ$. We weight this cost, $J_{KL}\left(p_{\ell }\right)$ for each ℓ∈ℒ according to what fraction of the overall mass in transformed atlas this distribution contributes to, with most weight attributed to larger atlas regions that correspondingly map to areas of high target density and lowest weight attributed to smaller atlas regions and those mapping to areas of low target density:

(20) $$J_{KL}\left(p_{\ell }\right)≐\frac{M_{\ell }^{A}}{\sum_{f\in ℱ}M_{f}^{T}}\sum_{f\in ℱ}p_{\ell }\left(f\right)\log\left(\frac{\bar{p_{\ell }}\left(f\right)}{\frac{1}{\left|ℱ\right|}}\right),$$

with $M_{\ell }^{A}$, the total atlas mass of region ℓ following deformation and $M_{f}^{T}$, the total target mass of feature f, computed in each case as the sum over the entire image varifold object.

We use LBFGS to optimize these pairs of momenta per particle together with the distributions ${\left(p_{\ell }\right)}_{\ell \in ℒ}$ and bandwith λ, for controlling the censoring function, so as to minimize the cost as given in (14), with the regularization on $v_{t}$ re-parameterized with the Hamiltonian:

(21) $$H\left(q,\rho ,v\right)+\sum_{\ell \in ℒ}J_{KL}\left(p_{\ell }\right)+{\left\|\varphi_{1}^{v}\cdot \mu_{A}^{p,\lambda }-\mu_{T}\right\|}_{M}^{2}.$$

### 4.5 Mutual Information Scoring for Feature Selection

To deduce which features are spatially variant, we assign to each feature a score based on mutual information. This score aims to capture not global variance in a feature distribution, but rather, within a local neighborhood, whether the spatial distribution of a given feature value (e.g. gene, cell type) is organized along particular boundaries. We simplify these boundaries to be vertical or horizontal lines only, and use mutual information to deduce how closely the distributions of feature values organize along these boundaries within local neighborhoods, sized according to a chosen scale.

In detail, this score specifically measures the mutual information between a random variable, $M^{g}$, that reflects the number of counts of feature g (e.g. a gene type, a cell type) in a given neighborhood, and a random variable, X, that partitions this neighborhood vertically or horizontally into two domains. We describe, here, a method for computing this score particularly in settings of large amounts of data, where discretization is favorable for computational efficiency. As an example, we specifically describe the setting of serial tissue sections measured for gene expression, as with MERFISH or BARseq. The method, as illustrated in Supplementary Figure 1, is applied for each gene independently on each measured section of tissue, where collective scores per gene can be garnered by tallying each gene’s score per section across the entire set of sections.

The support of the tissue section is first covered by a grid, as shown in the left panel of Supplementary Figure 1, with squares of size σ×σ. In the results shown in Section 2.6, we choose σ=50μm. In each square, we compute the total number of mRNA expressed per each gene in that square, denoted by $N^{g}$ for gene g. Let $F^{g}(t)=P\left(N^{g}\leq t\right)$ be the cumulative distribution function for gene g, estimated from the empirical distribution of $N^{g}$ across all squares in our grid. We define the binning function $\phi^{g}(n)=\sum_{k=1}^{q}1_{n\geq t_{k}}$ for $t_{k}=inf\left\{t\geq 0|F^{g}(t)\geq k/q\right\}$ and with k∈[1,q] denoting the k-th q-quantile. This gives a discrete (normalized) value of mRNA counts for gene g in each square of the grid, as shown in the middle panel of Supplementary Figure 1 for g=Gfap.

We define our discrete neighborhoods as megasquares, denoted ${\left(Q_{c}\right)}_{c\in 𝒞}$, with each comprised of a continguous set of 2K×2K grid squares. We consider all possible megasquares that can be defined across the grid, and index the squares within each megasquare by column index i=1,…,2K and row index j=1,…,2K, giving $Q_{c}={⋃}_{(i,j)\in {\left\{1,\cdots ,2K\right\}}^{2}}\;Q_{c,i,j}$. Finally, we define two partitioning schemes, denoted ↕ and ↔, corresponding to the partitioning of a megasquare into two equal vertical or two equal horizontal domains, with each domain in each scheme containing $2K^{2}$ squares. The right panel of Figure 1 shows a sample of 4 megasquares from the entire set ${\left(Q_{c}\right)}_{c\in C}$ that cover the grid.

The random variables of interest, X and $M^{g}$ are specified as functions of ω=(c,i,j,d)∈Ω with $\Omega =𝒞\times {\left[1,\;2K\right]}^{2}\times \{↕,↔\}$, the set of all possible selections of megasquare, square within the megasquare, and partitioning of the megasquare. Specifically, we denote C(ω)=c, the index of the megasquare, $N^{g}(\omega )$ the counts of gene g for the square $Q_{c,i,j}$ in megasquare, c, giving $M^{g}(\omega )=\phi \left(N^{g}(\omega )\right)\in [1,q]$, the q-quantile of the gene count, and X(ω)∈{b,t,l,r}, the partition $Q_{c,i,j}$ belongs to, dictated by direction d in ω as:

(22) $$X(\omega )=\left\{\begin{array}{ll} l & \text{if}\;d=↔,i\leq K\\ r & \text{if}\;d=↔,i>K\\ b & \text{if}\;d=↕,j\leq K\\ t & \text{if}\;d=↕,\;j>K \end{array}\right.$$

Choice of ω is made uniformly, with $P=\frac{1}{\left|\Omega \right|}\sum_{\omega \in \Omega }\delta_{\omega }$. Our score is thus, the conditional mutual information between X and $M^{g}$ given C:

(23) $$I\left(X;M^{g}|C\right)=\;\;\sum_{c,x,m}P\left(X=x,M^{g}=m,C=c\right)log\left(\frac{P\left(X\;=\;x,\;M^{g}\;=\;m|C\;=\;c\right)}{P(X\;=\;x|C\;=\;c)P\left(M^{g}\;=\;m|C\;=\;c\right)}\right)$$

### 4.6 Optimization Based Resampling Algorithm for Multi-Scale Representation

Section 2.5 introduced the scale-space resampling method for generating data approximations at different scales. We generate a hierarchy of approximation measures $\mu_{\sigma },\;\sigma_{1}>\sigma_{2}\ldots$ with increasing complexity as we descend in scale, $\left|I_{\sigma_{1}}\right|<\left|I_{\sigma_{2}}\right|<\ldots .$ The Algorithm 1 describes the generation of this reduced set of particles at scale, σ, where the physical locations, ${\tilde{x}}_{i}$, mass weights, ${\tilde{w}}_{i}$, and conditional probability distributions, ${\tilde{p}}_{i}$ for each particle in our reduced set $i\in \tilde{I}$ are optimized according to (6).

An implementation of Algorithm 1 utilizing LBFGS from PyTorch can be found at https://github.com/kstouff4/VarifoldApproximation/. The implementation was developed and tested on a single Quadro RTX 8000 GPU with 48GB memory. To increase speed efficiency, particles in both the high resolution and reduced set were first grouped into cubes and ordered in memory according to physical location. Total kernel operations needed in each optimization scheme were reduced by relegating all those between particles in cubes whose centers were at a distance greater than threshold (e.g. 4σ) a value of 0 without active computation. To reduce active memory load, larger datasets were halved or quartered along axes of physical space, with each section within a stack of sections aggregated into a reduced set of particles independently. The resulting reduced particle sets for each fraction were rejoined after optimization into a single image varifold object.

**Algorithm 1:** 

1.	Define lattice spanning support of data, with cubes $\propto \sigma^{3}$.
2.	For each cube containing at least one high resolution particle:
	(a) Select one particle, i∈I in the cube at random.
	(b) Initialize a particle, $\tilde{i}\in \tilde{I}$, as ${\tilde{w}}_{i}\delta_{x_{i}}⊗p^{u}$, where ${\tilde{w}}_{i}=\sum_{j}w_{j}$ for all particles, j, within the cube and $p^{u}(f)=\frac{1}{\left|ℱ\right|}\forall f\in ℱ$ (the uniform distribution).
**Single Optimization:**	
1.	Fix $\tilde{x_{i}}\;\forall i\in \tilde{I}$.
2.	Minimize (6) with respect to all ${\tilde{w}}_{i},\;{\tilde{p}}_{i}$ with LBFGS for 200 iterations.
**Joint Optimization:**	
1.	Minimize (6) with respect to all ${\tilde{x}}_{i},\;{\tilde{w}}_{i},\;{\tilde{p}}_{i}$ with LBFGS for 200 iterations.

### 4.7 Estimating the Censoring Function

For brains with censored planes corresponding to the rostral-caudal directions only (e.g. whole coronal sections), we apply a censoring function oriented only to the rostral-caudal axis. This function of space is given in molecular target coordinates as the sum of two hyperbolic tangent functions, giving for a location $x\in R^{3}$:

(24) $$\alpha_{\lambda }(x)≐\frac{1}{2}tanh\left(\frac{\left\langle x\;-\;a_{0},\;n_{0}\right\rangle }{\lambda }\right)+tanh\left(\frac{\left\langle x\;-\;a_{1},\;n_{1}\right\rangle }{\lambda }\right)$$

with $a_{0},\;a_{1}$ two points in the first and last coronal sections of the molecular data, respectively, and $n_{0},\;n_{1}$ the respective normal vectors to the plane of each section, pointing towards the interior sections of the dataset. Support weights, $\alpha_{\lambda }(x)$ are thus within the range, $\left[0,\;1\right]\;\forall x\in R^{3}$, with $\alpha_{\lambda }(x)=1$ indicating the support of the molecular dataset and 0 outside the support, with the bandwith λ controlling the width of the transition zone from inside to outside the support with $0<\alpha_{\lambda }(x)<1$. We estimate λ jointly with geometric transformations and feature distributions, with added regularization cost:

(25) $$J_{s}(\lambda )≐\frac{\lambda^{2}}{0.1}ln\left(\frac{\lambda^{2}}{0.1}\right)+1-\frac{\lambda^{2}}{0.1}.$$

In the setting of stacks of hemibrain sections, as described in [15], the support weights necessarily capture boundaries around the target measured dataset at rostral, caudal, and medial edges. The medial boundary typically varies along the rostral-caudal axis with differences in the extent of tissue capture per section measured. To accommodate this irregularity with a smooth transition function from the interior to exterior of the support, we take the support weight function, $\alpha_{\lambda }$, to be the output of a UNET trained to delineate interior support from exterior in the target physical space.

Training data is generated at a scale of 200*μ*m, where high resolution particle measures have been aggregated into a reduced set of particle measures, of size *o*(1000) per section (see Section 2.5). To each section, 15–20 particles are placed manually along the medial boundary. A label of 1 is assigned to all particles in the aggregation and 0 to those particles manually added to each section. The particles per section are stacked according to given spacing (e.g. 200*μ*m) and treated as a single 3D object for estimation of the support. Added particles with label 0 are weighted 1000 : 1 to balance the discrepancy in numbers of particles contained within versus outside the support.

Here, we use the NeuralNetClassifier from skorch to implement a 3 layer neural network. Two linear layers, sized 3 × 15 and 15 × 5, respectively are each followed by an Exponential Linear Unit. The final output linear layer, sized 5 × 1, is smoothed with a hyperbolic tangent, analogous to the setting of single axis weights described above (see Section 4.7) and shifted and rescaled to be in the interval [0, 1]. As in (25), a bandwith parameter, λ, is estimated in tandem with geometric and feature transformations, controlling the width of the transition zone from interior to exterior of the support.

## Supplementary Material

Supplement 1

## Data Availability

Serial MERFISH sections from the Allen Institute were produced under the BRAIN Initiative Cell Census Network (BICCN, www.biccn.org, RRID:SCR 015820) and are available at the Brain Image Library (BIL, https://www.brainimagelibrary.org/index.html) under doi https://doi.org/10.35077/g.610. Serial BARseq sections with cell-level data are available at Mendeley data (doi; 10.17632/8bhhk7c5n9.1). The Allen CCFv3 used in this study is available at https://download.alleninstitute.org/informatics-archive/current-release/mouse_ccf/annotation/ccf_2022/.
