# Supplementary material for: xIV-LDDMM Toolkit: A Suite of Image-Varifold Based Technologies for Representing and Mapping 3D Imaging and Spatial-omics Data Simultaneously Across Scales
Source: bioRxiv. 2024 Nov 5:2024.11.04.621983. Preprint. [Version 1] doi: 10.1101/2024.11.04.621983 (PMC11580965; doi:10.1101/2024.11.04.621983)
Supplement: Supplementary file 1 [file NIHPP2024.11.04.621983v1-supplement-1.pdf]

bioRxiv preprint doi: https://doi.org/10.1101/2024.11.04.621983; this version posted November 5, 2024. The copyright holder for this preprint (which was not certified by peer review) is the author/funder, who has granted bioRxiv a license to display the preprint in perpetuity. It is made available under aCC-BY 4.0 International license.

Supplementary Figures and Tables

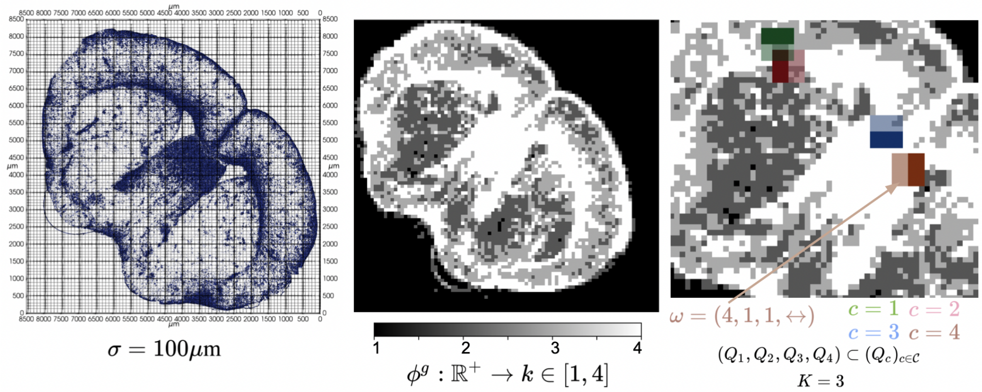

Fig. 1 Steps in computing mutual information score for each gene in each tissue section of MERFISH dataset [2, 3]. Left shows raw data as individual mRNA reads for gene g = *Gfap*. The support of the tissue is covered by a grid with squares of size $\sigma \times \sigma$, with $\sigma = 100\mu$m shown here. Middle shows output of binning function, ϕ^g on the counts of gene g in each grid square, with $q = 4$. Right shows zoomed in portion of tissue with sample of 4 megacubes out of the entire set $(Q_c)_{c\in\mathcal{C}}$. Example ω given for the individual grid square located at the bottom left corner of Q_4.

bioRxiv preprint doi: https://doi.org/10.1101/2024.11.04.621983; this version posted November 5, 2024. The copyright holder for this preprint (which was not certified by peer review) is the author/funder, who has granted bioRxiv a license to display the preprint in perpetuity. It is made available under aCC-BY 4.0 International license.

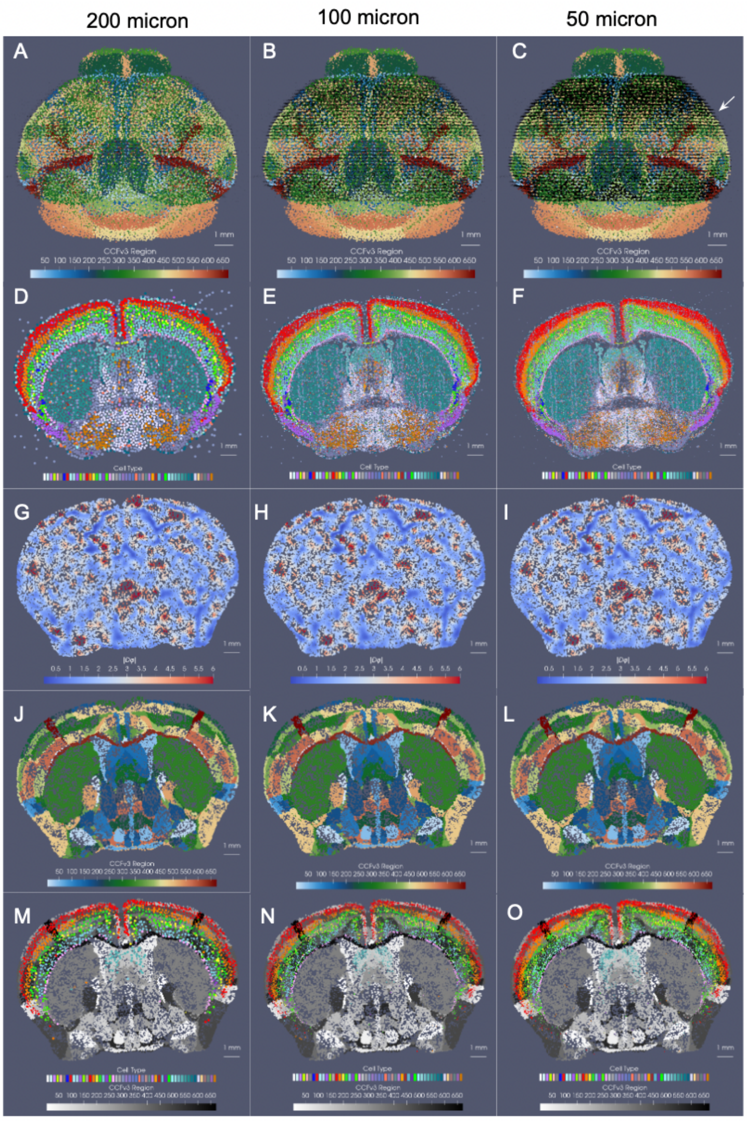

Fig. 2 Results for mapping 200 μm CCFv3 to different scales of BARseq whole brain sections (200, 100, 50 μm, estimated with scale-space resampling as described in Section 2.5) with runtimes given in Supplementary Table 2. A-C show global alignment of Allen CCFv3 to BARseq target at each resolution (particle measures shown in black). D-F show a single BARseq section (white arrow in C) from the optimized aggregation at each scale, with cell type carrying highest probability for each particle delineated by color. G-I show section of transformed CCFv3 intersecting given slice of BARseq target, with determinant of the Jacobian of the diffeomorphism, $|D\varphi|$, exhibiting areas of expansion (red) and contraction (blue). J-L show section of transformed CCFv3 with initial atlas labels. M-O show section of transformed CCFv3 overlaid with target cells within the cortical layers and the lateral septal nucleus.

bioRxiv preprint doi: https://doi.org/10.1101/2024.11.04.621983; this version posted November 5, 2024. The copyright holder for this preprint (which was not certified by peer review) is the author/funder, who has granted bioRxiv a license to display the preprint in perpetuity. It is made available under aCC-BY 4.0 International license.

Method	Scale-Space Resampling	K Means	Nearest Neighbor Grid Resampling	Gaussian Smoothing Grid Resampling
Number of Particles	21,317	20,000	16,324	16,324
$\|\mu_\sigma - \mu\|_M^2$	142,336	1,517,312	9,027,968	77,743,872

Table 1 Comparison of scale-space resampling to alternative methods of resampling with comparable number of particles for section of BARseq measured tissue with 16 selected genes. Varifold normed distance measures similarity to high resolution target.

Resolution	# Particles	# Features	Runtime (hr)	Memory (MB)
200 μm	163491	23	18.5	17.66
200 μm	163491	29	20.75	21.58
200 μm	163491	52	20.67	36.62
100 μm	583776	52	20.58	130.77
50 μm	1705989	52	24.65	382.14

Table 2 Efficiency metrics of xIV-LDDMM for mapping 200 μm representation of Allen CCFv3 to a stack of 40 whole brain BARseq sections. Runtime is computed as total time to run 150 iterations of optimization scheme for estimating geometric transformation and feature distributions. Memory is tallied as the amount of storage per target dataset given as .pt file. Resolution, # particles, and # features are all given for target image varifold. Runtime measures were computed on an NVIDIA RTX A5000 GPU.

	L2/3	L4	L5	L6
1	0.81	0.61	0.75	0.71
2	0.84	0.60	0.73	0.69
3	0.73	0.52	0.75	0.73
4	0.83	0.60	0.74	0.70
5	0.77	0.53	0.70	0.67
6	0.79	0.55	0.75	0.72
7	0.79	0.57	0.75	0.68

Table 3 Fraction of correctly aligned cells per layer in 7 BARseq hemi-brain samples manually aligned to CCFv3. Averages across layer designations are $0.79 \pm 0.04, 0.57 \pm 0.04, 0.74 \pm 0.02, 0.70 \pm 0.02$.
